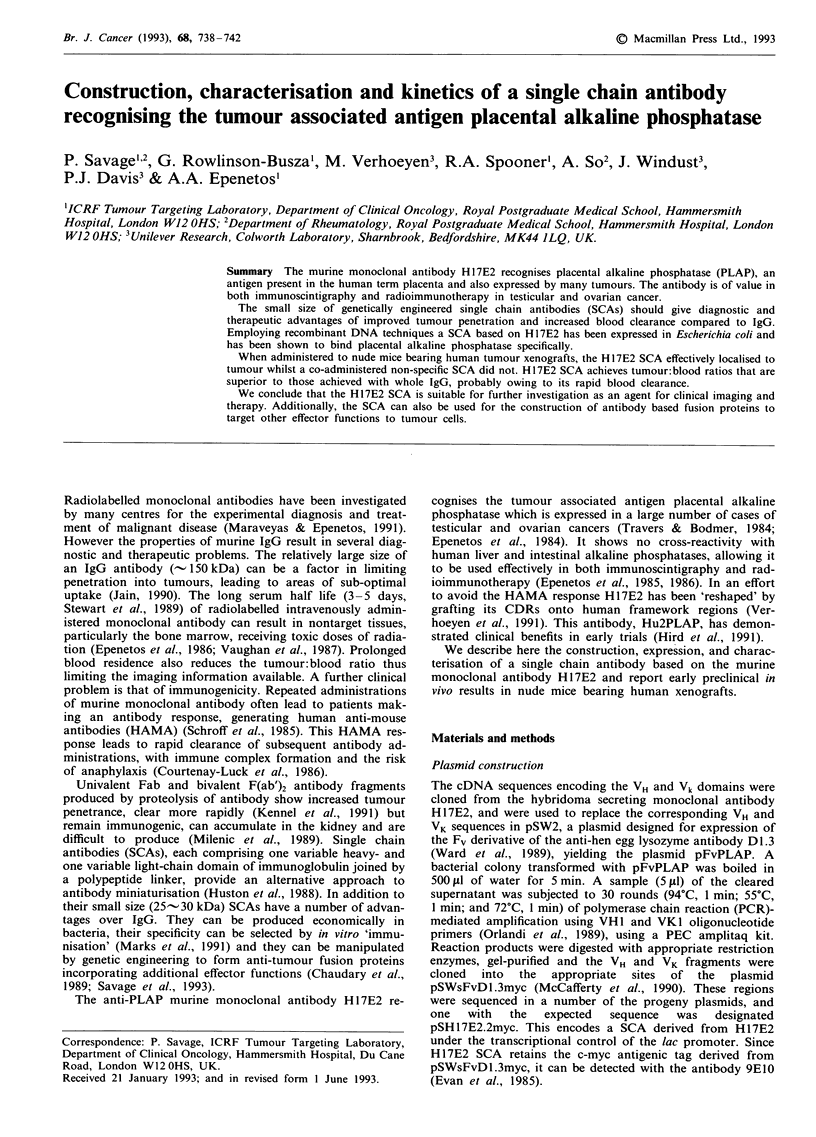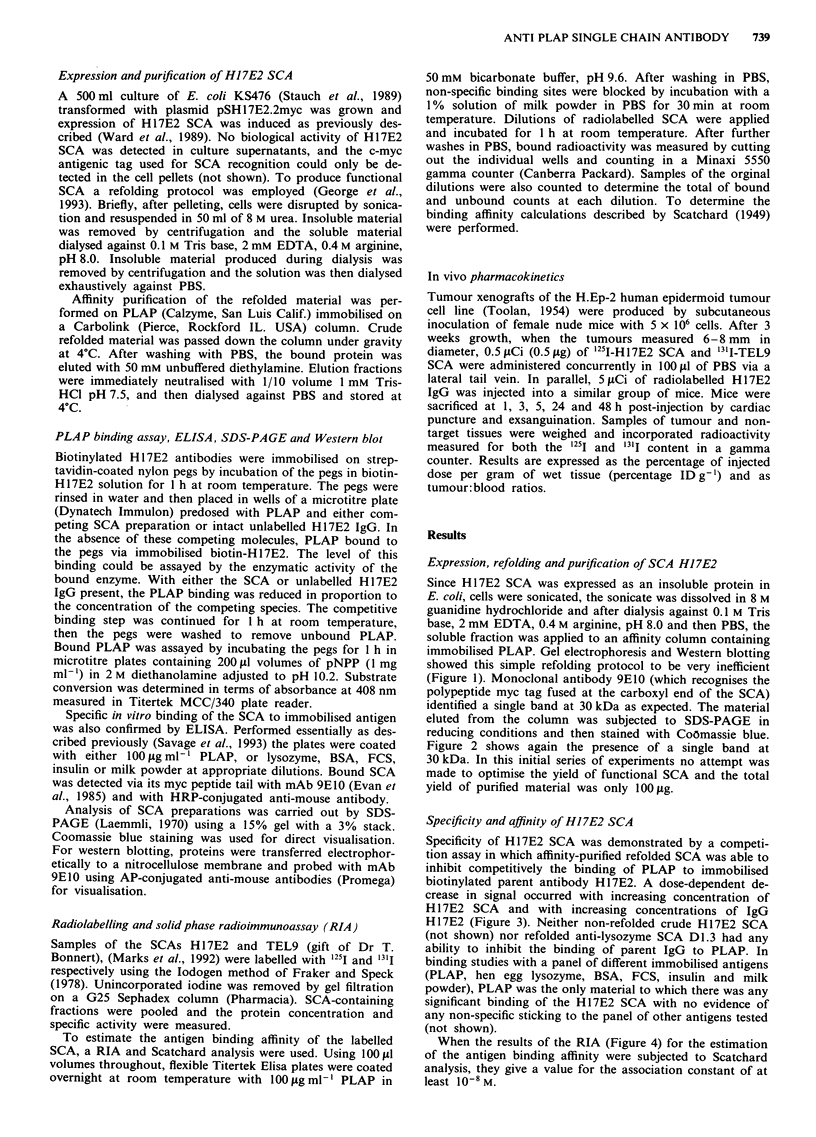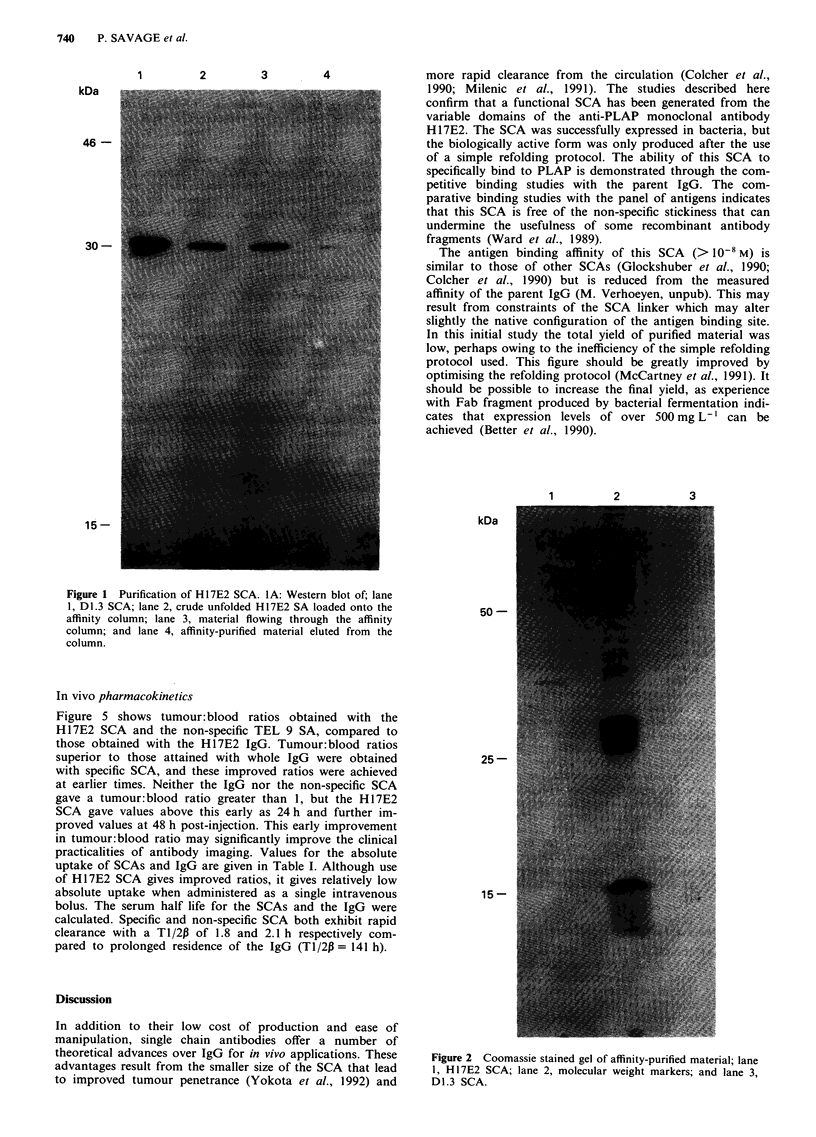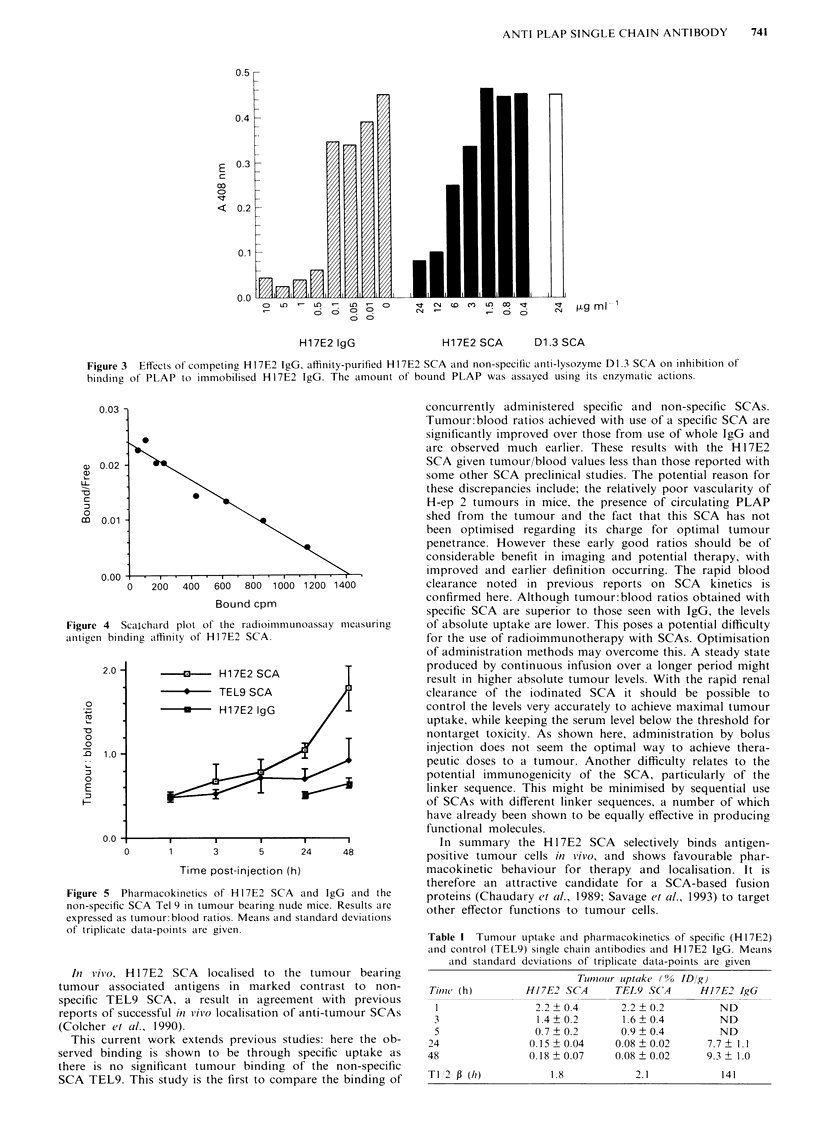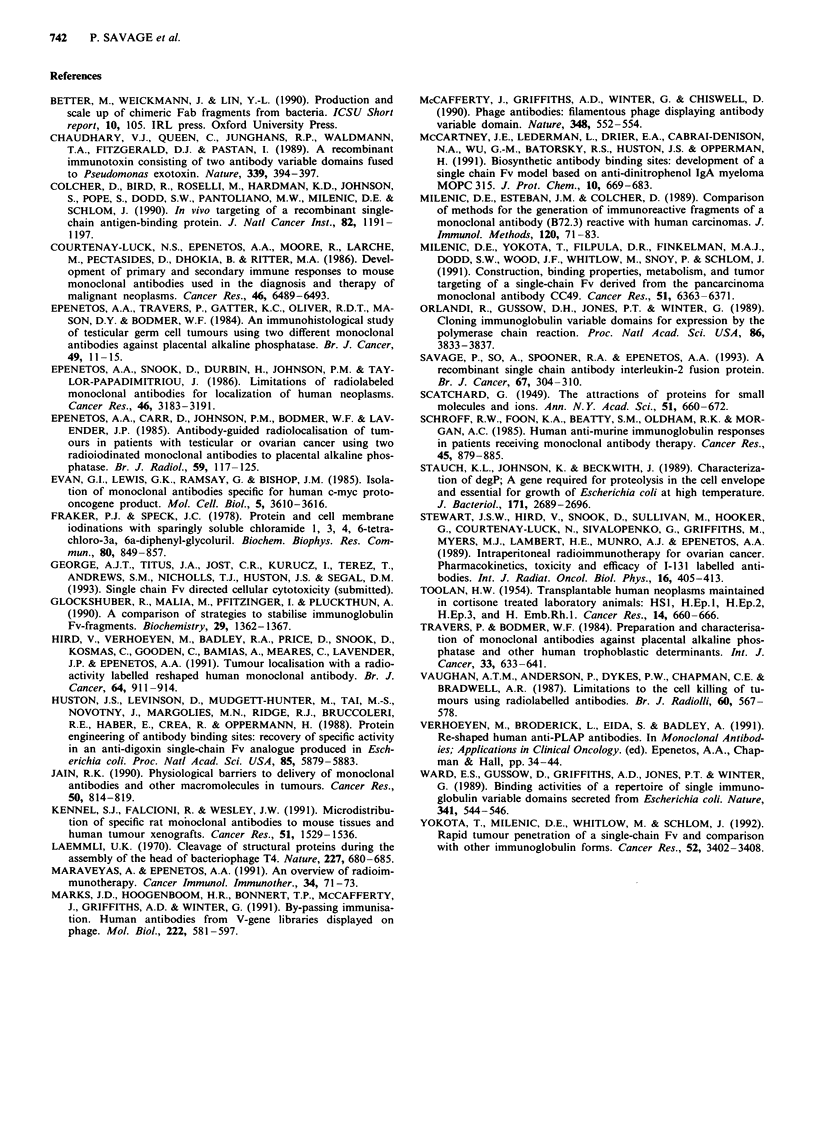# Construction, characterisation and kinetics of a single chain antibody recognising the tumour associated antigen placental alkaline phosphatase.

**DOI:** 10.1038/bjc.1993.420

**Published:** 1993-10

**Authors:** P. Savage, G. Rowlinson-Busza, M. Verhoeyen, R. A. Spooner, A. So, J. Windust, P. J. Davis, A. A. Epenetos

**Affiliations:** Department of Clinical Oncology, Royal Postgraduate Medical School, Hammersmith Hospital, London, UK.

## Abstract

**Images:**


Br. J. Cancer (1993), 68, 738-742 © Macmillan Press Ltd., 1993

### Construction, characterisation and kinetics of a single chain antibody recognising the tumour associated antigen placental alkaline phosphatase

P. Savage[1,2], G. Rowlinson-Busza[1], M. Verhoeyen[3], R.A. Spooner[1], A. So[2], J. Windust[3], P.J. Davis[3] & A.A. Epenetos[1]

[1]*ICRF Tumour Targeting Laboratory, Department of Clinical Oncology, Royal Postgraduate Medical School, Hammersmith Hospital, London W12 0HS;* [2]*Department of Rheumatology, Royal Postgraduate Medical School, Hammersmith Hospital, London W12 0HS;* [3]*Unilever Research, Colworth Laboratory, Sharnbrook, Bedfordshire, MK44 1LQ, UK.*

**Summary** The murine monoclonal antibody H17E2 recognises placental alkaline phosphatase (PLAP), an antigen present in the human term placenta and also expressed by many tumours. The antibody is of value in both immunoscintigraphy and radioimmunotherapy in testicular and ovarian cancer.

The small size of genetically engineered single chain antibodies (SCAs) should give diagnostic and therapeutic advantages of improved tumour penetration and increased blood clearance compared to IgG. Employing recombinant DNA techniques a SCA based on H17E2 has been expressed in *Escherichia coli* and has been shown to bind placental alkaline phosphatase specifically.

When administered to nude mice bearing human tumour xenografts, the H17E2 SCA effectively localised to tumour whilst a co-administered non-specific SCA did not. H17E2 SCA achieves tumour:blood ratios that are superior to those achieved with whole IgG, probably owing to its rapid blood clearance.

We conclude that the H17E2 SCA is suitable for further investigation as an agent for clinical imaging and therapy. Additionally, the SCA can also be used for the construction of antibody based fusion proteins to target other effector functions to tumour cells.

Radiolabelled monoclonal antibodies have been investigated by many centres for the experimental diagnosis and treatment of malignant disease (Maraveyas & Epenetos, 1991). However the properties of murine IgG result in several diagnostic and therapeutic problems. The relatively large size of an IgG antibody (~150 kDa) can be a factor in limiting penetration into tumours, leading to areas of sub-optimal uptake (Jain, 1990). The long serum half life (3-5 days, Stewart *et al.*, 1989) of radiolabelled intravenously administered monoclonal antibody can result in nontarget tissues, particularly the bone marrow, receiving toxic doses of radiation (Epenetos *et al.*, 1986; Vaughan *et al.*, 1987). Prolonged blood residence also reduces the tumour:blood ratio thus limiting the imaging information available. A further clinical problem is that of immunogenicity. Repeated administrations of murine monoclonal antibody often lead to patients making an antibody response, generating human anti-mouse antibodies (HAMA) (Schroff *et al.*, 1985). This HAMA response leads to rapid clearance of subsequent antibody administrations, with immune complex formation and the risk of anaphylaxis (Courtenay-Luck *et al.*, 1986).

Univalent Fab and bivalent F(ab')$_2$ antibody fragments produced by proteolysis of antibody show increased tumour penetrance, clear more rapidly (Kennel *et al.*, 1991) but remain immunogenic, can accumulate in the kidney and are difficult to produce (Milenic *et al.*, 1989). Single chain antibodies (SCAs), each comprising one variable heavy- and one variable light-chain domain of immunoglobulin joined by a polypeptide linker, provide an alternative approach to antibody miniaturisation (Huston *et al.*, 1988). In addition to their small size (25~30 kDa) SCAs have a number of advantages over IgG. They can be produced economically in bacteria, their specificity can be selected by *in vitro* 'immunisation' (Marks *et al.*, 1991) and they can be manipulated by genetic engineering to form anti-tumour fusion proteins incorporating additional effector functions (Chaudary *et al.*, 1989; Savage *et al.*, 1993).

The anti-PLAP murine monoclonal antibody H17E2 recognises the tumour associated antigen placental alkaline phosphatase which is expressed in a large number of cases of testicular and ovarian cancers (Travers & Bodmer, 1984; Epenetos *et al.*, 1984). It shows no cross-reactivity with human liver and intestinal alkaline phosphatases, allowing it to be used effectively in both immunoscintigraphy and radioimmunotherapy (Epenetos *et al.*, 1985, 1986). In an effort to avoid the HAMA response H17E2 has been 'reshaped' by grafting its CDRs onto human framework regions (Verhoeyen *et al.*, 1991). This antibody, Hu2PLAP, has demonstrated clinical benefits in early trials (Hird *et al.*, 1991).

We describe here the construction, expression, and characterisation of a single chain antibody based on the murine monoclonal antibody H17E2 and report early preclinical *in vivo* results in nude mice bearing human xenografts.

### Materials and methods

### *Plasmid construction*

The cDNA sequences encoding the $V_H$ and $V_k$ domains were cloned from the hybridoma secreting monoclonal antibody H17E2, and were used to replace the corresponding $V_H$ and $V_K$ sequences in pSW2, a plasmid designed for expression of the $F_V$ derivative of the anti-hen egg lysozyme antibody D1.3 (Ward *et al.*, 1989), yielding the plasmid pFvPLAP. A bacterial colony transformed with pFvPLAP was boiled in 500 µl of water for 5 min. A sample (5 µl) of the cleared supernatant was subjected to 30 rounds (94°C, 1 min; 55°C, 1 min; and 72°C, 1 min) of polymerase chain reaction (PCR)-mediated amplification using VH1 and VK1 oligonucleotide primers (Orlandi *et al.*, 1989), using a PEC amplitaq kit. Reaction products were digested with appropriate restriction enzymes, gel-purified and the $V_H$ and $V_K$ fragments were cloned into the appropriate sites of the plasmid pSWsFvD1.3myc (McCafferty *et al.*, 1990). These regions were sequenced in a number of the progeny plasmids, and one with the expected sequence was designated pSH17E2.2myc. This encodes a SCA derived from H17E2 under the transcriptional control of the *lac* promoter. Since H17E2 SCA retains the c-myc antigenic tag derived from pSWsFvD1.3myc, it can be detected with the antibody 9E10 (Evan *et al.*, 1985).

Correspondence: P. Savage, ICRF Tumour Targeting Laboratory, Department of Clinical Oncology, Hammersmith Hospital, Du Cane Road, London W12 0HS, UK.
Received 21 January 1993; and in revised form 1 June 1993.

*Expression and purification of H17E2 SCA*

A 500 ml culture of *E. coli* KS476 (Stauch *et al.*, 1989) transformed with plasmid pSH17E2.2myc was grown and expression of H17E2 SCA was induced as previously described (Ward *et al.*, 1989). No biological activity of H17E2 SCA was detected in culture supernatants, and the c-myc antigenic tag used for SCA recognition could only be detected in the cell pellets (not shown). To produce functional SCA a refolding protocol was employed (George *et al.*, 1993). Briefly, after pelleting, cells were disrupted by sonication and resuspended in 50 ml of 8 M urea. Insoluble material was removed by centrifugation and the soluble material dialysed against 0.1 M Tris base, 2 mM EDTA, 0.4 M arginine, pH 8.0. Insoluble material produced during dialysis was removed by centrifugation and the solution was then dialysed exhaustively against PBS.

Affinity purification of the refolded material was performed on PLAP (Calzyme, San Luis Calif.) immobilised on a Carbolink (Pierce, Rockford IL. USA) column. Crude refolded material was passed down the column under gravity at 4°C. After washing with PBS, the bound protein was eluted with 50 mM unbuffered diethylamine. Elution fractions were immediately neutralised with 1/10 volume 1 mM Tris-HCl pH 7.5, and then dialysed against PBS and stored at 4°C.

*PLAP binding assay, ELISA, SDS-PAGE and Western blot*

Biotinylated H17E2 antibodies were immobilised on streptavidin-coated nylon pegs by incubation of the pegs in biotin-H17E2 solution for 1 h at room temperature. The pegs were rinsed in water and then placed in wells of a microtitre plate (Dynatech Immulon) predosed with PLAP and either competing SCA preparation or intact unlabelled H17E2 IgG. In the absence of these competing molecules, PLAP bound to the pegs via immobilised biotin-H17E2. The level of this binding could be assayed by the enzymatic activity of the bound enzyme. With either the SCA or unlabelled H17E2 IgG present, the PLAP binding was reduced in proportion to the concentration of the competing species. The competitive binding step was continued for 1 h at room temperature, then the pegs were washed to remove unbound PLAP. Bound PLAP was assayed by incubating the pegs for 1 h in microtitre plates containing 200 µl volumes of pNPP (1 mg $ml^{-1}$) in 2 M diethanolamine adjusted to pH 10.2. Substrate conversion was determined in terms of absorbance at 408 nm measured in Titertek MCC/340 plate reader.

Specific *in vitro* binding of the SCA to immobilised antigen was also confirmed by ELISA. Performed essentially as described previously (Savage *et al.*, 1993) the plates were coated with either 100 $\mu g\ ml^{-1}$ PLAP, or lysozyme, BSA, FCS, insulin or milk powder at appropriate dilutions. Bound SCA was detected via its myc peptide tail with mAb 9E10 (Evan *et al.*, 1985) and with HRP-conjugated anti-mouse antibody.

Analysis of SCA preparations was carried out by SDS-PAGE (Laemmli, 1970) using a 15% gel with a 3% stack. Coomassie blue staining was used for direct visualisation. For western blotting, proteins were transferred electrophoretically to a nitrocellulose membrane and probed with mAb 9E10 using AP-conjugated anti-mouse antibodies (Promega) for visualisation.

*Radiolabelling and solid phase radioimmunoassay (RIA)*

Samples of the SCAs H17E2 and TEL9 (gift of Dr T. Bonnert), (Marks *et al.*, 1992) were labelled with $^{125}I$ and $^{131}I$ respectively using the Iodogen method of Fraker and Speck (1978). Unincorporated iodine was removed by gel filtration on a G25 Sephadex column (Pharmacia). SCA-containing fractions were pooled and the protein concentration and specific activity were measured.

To estimate the antigen binding affinity of the labelled SCA, a RIA and Scatchard analysis were used. Using 100 µl volumes throughout, flexible Titertek Elisa plates were coated overnight at room temperature with 100 $\mu g\ ml^{-1}$ PLAP in 50 mM bicarbonate buffer, pH 9.6. After washing in PBS, non-specific binding sites were blocked by incubation with a 1% solution of milk powder in PBS for 30 min at room temperature. Dilutions of radiolabelled SCA were applied and incubated for 1 h at room temperature. After further washes in PBS, bound radioactivity was measured by cutting out the individual wells and counting in a Minaxi 5550 gamma counter (Canberra Packard). Samples of the orginal dilutions were also counted to determine the total of bound and unbound counts at each dilution. To determine the binding affinity calculations described by Scatchard (1949) were performed.

In vivo *pharmacokinetics*

Tumour xenografts of the H.Ep-2 human epidermoid tumour cell line (Toolan, 1954) were produced by subcutaneous inoculation of female nude mice with $5 \times 10^6$ cells. After 3 weeks growth, when the tumours measured 6–8 mm in diameter, 0.5 µCi (0.5 µg) of $^{125}I$-H17E2 SCA and $^{131}I$-TEL9 SCA were administered concurrently in 100 µl of PBS via a lateral tail vein. In parallel, 5 µCi of radiolabelled H17E2 IgG was injected into a similar group of mice. Mice were sacrificed at 1, 3, 5, 24 and 48 h post-injection by cardiac puncture and exsanguination. Samples of tumour and non-target tissues were weighed and incorporated radioactivity measured for both the $^{125}I$ and $^{131}I$ content in a gamma counter. Results are expressed as the percentage of injected dose per gram of wet tissue (percentage ID $g^{-1}$) and as tumour:blood ratios.

### Results

*Expression, refolding and purification of SCA H17E2*

Since H17E2 SCA was expressed as an insoluble protein in *E. coli*, cells were sonicated, the sonicate was dissolved in 8 M guanidine hydrochloride and after dialysis against 0.1 M Tris base, 2 mM EDTA, 0.4 M arginine, pH 8.0 and then PBS, the soluble fraction was applied to an affinity column containing immobilised PLAP. Gel electrophoresis and Western blotting showed this simple refolding protocol to be very inefficient (Figure 1). Monoclonal antibody 9E10 (which recognises the polypeptide myc tag fused at the carboxyl end of the SCA) identified a single band at 30 kDa as expected. The material eluted from the column was subjected to SDS-PAGE in reducing conditions and then stained with Coömassie blue. Figure 2 shows again the presence of a single band at 30 kDa. In this initial series of experiments no attempt was made to optimise the yield of functional SCA and the total yield of purified material was only 100 µg.

*Specificity and affinity of H17E2 SCA*

Specificity of H17E2 SCA was demonstrated by a competition assay in which affinity-purified refolded SCA was able to inhibit competitively the binding of PLAP to immobilised biotinylated parent antibody H17E2. A dose-dependent decrease in signal occurred with increasing concentration of H17E2 SCA and with increasing concentrations of IgG H17E2 (Figure 3). Neither non-refolded crude H17E2 SCA (not shown) nor refolded anti-lysozyme SCA D1.3 had any ability to inhibit the binding of parent IgG to PLAP. In binding studies with a panel of different immobilised antigens (PLAP, hen egg lysozyme, BSA, FCS, insulin and milk powder), PLAP was the only material to which there was any significant binding of the H17E2 SCA with no evidence of any non-specific sticking to the panel of other antigens tested (not shown).

When the results of the RIA (Figure 4) for the estimation of the antigen binding affinity were subjected to Scatchard analysis, they give a value for the association constant of at least $10^{-8}$ M.

**Figure 1** Purification of H17E2 SCA. 1A: Western blot of; lane 1, D1.3 SCA; lane 2, crude unfolded H17E2 SA loaded onto the affinity column; lane 3, material flowing through the affinity column; and lane 4, affinity-purified material eluted from the column.

### In vivo *pharmacokinetics*

Figure 5 shows tumour:blood ratios obtained with the H17E2 SCA and the non-specific TEL 9 SA, compared to those obtained with the H17E2 IgG. Tumour:blood ratios superior to those attained with whole IgG were obtained with specific SCA, and these improved ratios were achieved at earlier times. Neither the IgG nor the non-specific SCA gave a tumour:blood ratio greater than 1, but the H17E2 SCA gave values above this early as 24 h and further improved values at 48 h post-injection. This early improvement in tumour:blood ratio may significantly improve the clinical practicalities of antibody imaging. Values for the absolute uptake of SCAs and IgG are given in Table I. Although use of H17E2 SCA gives improved ratios, it gives relatively low absolute uptake when administered as a single intravenous bolus. The serum half life for the SCAs and the IgG were calculated. Specific and non-specific SCA both exhibit rapid clearance with a $T1/2\beta$ of 1.8 and 2.1 h respectively compared to prolonged residence of the IgG ($T1/2\beta = 141$ h).

### Discussion

In addition to their low cost of production and ease of manipulation, single chain antibodies offer a number of theoretical advances over IgG for *in vivo* applications. These advantages result from the smaller size of the SCA that lead to improved tumour penetrance (Yokota *et al.*, 1992) and more rapid clearance from the circulation (Colcher *et al.*, 1990; Milenic *et al.*, 1991). The studies described here confirm that a functional SCA has been generated from the variable domains of the anti-PLAP monoclonal antibody H17E2. The SCA was successfully expressed in bacteria, but the biologically active form was only produced after the use of a simple refolding protocol. The ability of this SCA to specifically bind to PLAP is demonstrated through the competitive binding studies with the parent IgG. The comparative binding studies with the panel of antigens indicates that this SCA is free of the non-specific stickiness that can undermine the usefulness of some recombinant antibody fragments (Ward *et al.*, 1989).

The antigen binding affinity of this SCA ($> 10^{-8}$ M) is similar to those of other SCAs (Glockshuber *et al.*, 1990; Colcher *et al.*, 1990) but is reduced from the measured affinity of the parent IgG (M. Verhoeyen, unpub). This may result from constraints of the SCA linker which may alter slightly the native configuration of the antigen binding site. In this initial study the total yield of purified material was low, perhaps owing to the inefficiency of the simple refolding protocol used. This figure should be greatly improved by optimising the refolding protocol (McCartney *et al.*, 1991). It should be possible to increase the final yield, as experience with Fab fragment produced by bacterial fermentation indicates that expression levels of over 500 mg $L^{-1}$ can be achieved (Better *et al.*, 1990).

**Figure 2** Coomassie stained gel of affinity-purified material; lane 1, H17E2 SCA; lane 2, molecular weight markers; and lane 3, D1.3 SCA.

**Figure 3** Effects of competing H17E2 IgG, affinity-purified H17E2 SCA and non-specific anti-lysozyme D1.3 SCA on inhibition of binding of PLAP to immobilised H17E2 IgG. The amount of bound PLAP was assayed using its enzymatic actions.

**Figure 4** Scatchard plot of the radioimmunoassay measuring antigen binding affinity of H17E2 SCA.

**Figure 5** Pharmacokinetics of H17E2 SCA and IgG and the non-specific SCA Tel 9 in tumour bearing nude mice. Results are expressed as tumour:blood ratios. Means and standard deviations of triplicate data-points are given.

*In vivo*, H17E2 SCA localised to the tumour bearing tumour associated antigens in marked contrast to non-specific TEL9 SCA, a result in agreement with previous reports of successful *in vivo* localisation of anti-tumour SCAs (Colcher *et al.*, 1990).

This current work extends previous studies: here the observed binding is shown to be through specific uptake as there is no significant tumour binding of the non-specific SCA TEL9. This study is the first to compare the binding of concurrently administered specific and non-specific SCAs. Tumour:blood ratios achieved with use of a specific SCA are significantly improved over those from use of whole IgG and are observed much earlier. These results with the H17E2 SCA given tumour/blood values less than those reported with some other SCA preclinical studies. The potential reason for these discrepancies include; the relatively poor vascularity of H-ep 2 tumours in mice, the presence of circulating PLAP shed from the tumour and the fact that this SCA has not been optimised regarding its charge for optimal tumour penetrance. However these early good ratios should be of considerable benefit in imaging and potential therapy, with improved and earlier definition occurring. The rapid blood clearance noted in previous reports on SCA kinetics is confirmed here. Although tumour:blood ratios obtained with specific SCA are superior to those seen with IgG, the levels of absolute uptake are lower. This poses a potential difficulty for the use of radioimmunotherapy with SCAs. Optimisation of administration methods may overcome this. A steady state produced by continuous infusion over a longer period might result in higher absolute tumour levels. With the rapid renal clearance of the iodinated SCA it should be possible to control the levels very accurately to achieve maximal tumour uptake, while keeping the serum level below the threshold for nontarget toxicity. As shown here, administration by bolus injection does not seem the optimal way to achieve therapeutic doses to a tumour. Another difficulty relates to the potential immunogenicity of the SCA, particularly of the linker sequence. This might be minimised by sequential use of SCAs with different linker sequences, a number of which have already been shown to be equally effective in producing functional molecules.

In summary the H17E2 SCA selectively binds antigen-positive tumour cells *in vivo*, and shows favourable pharmacokinetic behaviour for therapy and localisation. It is therefore an attractive candidate for a SCA-based fusion proteins (Chaudary *et al.*, 1989; Savage *et al.*, 1993) to target other effector functions to tumour cells.

**Table I** Tumour uptake and pharmacokinetics of specific (H17E2) and control (TEL9) single chain antibodies and H17E2 IgG. Means and standard deviations of triplicate data-points are given

| | *Tumour uptake (% ID/g)* | | |
|---|---|---|---|
| *Time* (h) | *H17E2 SCA* | *TEL9 SCA* | *H17E2 IgG* |
| 1 | 2.2 ± 0.4 | 2.2 ± 0.2 | ND |
| 3 | 1.4 ± 0.2 | 1.6 ± 0.4 | ND |
| 5 | 0.7 ± 0.2 | 0.9 ± 0.4 | ND |
| 24 | 0.15 ± 0.04 | 0.08 ± 0.02 | 7.7 ± 1.1 |
| 48 | 0.18 ± 0.07 | 0.08 ± 0.02 | 9.3 ± 1.0 |
| T1/2 β (*h*) | 1.8 | 2.1 | 141 |

### References

BETTER, M., WEICKMANN, J. & LIN, Y.-L. (1990). Production and scale up of chimeric Fab fragments from bacteria. *ICSU Short report*, **10**, 105. IRL press. Oxford University Press.

CHAUDHARY, V.J., QUEEN, C., JUNGHANS, R.P., WALDMANN, T.A., FITZGERALD, D.J. & PASTAN, I. (1989). A recombinant immunotoxin consisting of two antibody variable domains fused to *Pseudomonas* exotoxin. *Nature*, **339**, 394-397.

COLCHER, D., BIRD, R., ROSELLI, M., HARDMAN, K.D., JOHNSON, S., POPE, S., DODD, S.W., PANTOLIANO, M.W., MILENIC, D.E. & SCHLOM, J. (1990). *In vivo* targeting of a recombinant single-chain antigen-binding protein. *J. Natl Cancer Inst.*, **82**, 1191-1197.

COURTENAY-LUCK, N.S., EPENETOS, A.A., MOORE, R., LARCHE, M., PECTASIDES, D., DHOKIA, B. & RITTER, M.A. (1986). Development of primary and secondary immune responses to mouse monoclonal antibodies used in the diagnosis and therapy of malignant neoplasms. *Cancer Res.*, **46**, 6489-6493.

EPENETOS, A.A., TRAVERS, P., GATTER, K.C., OLIVER, R.D.T., MASON, D.Y. & BODMER, W.F. (1984). An immunohistological study of testicular germ cell tumours using two different monoclonal antibodies against placental alkaline phosphatase. *Br. J. Cancer*, **49**, 11-15.

EPENETOS, A.A., SNOOK, D., DURBIN, H., JOHNSON, P.M. & TAYLOR-PAPADIMITRIOU, J. (1986). Limitations of radiolabeled monoclonal antibodies for localization of human neoplasms. *Cancer Res.*, **46**, 3183-3191.

EPENETOS, A.A., CARR, D., JOHNSON, P.M., BODMER, W.F. & LAVENDER, J.P. (1985). Antibody-guided radiolocalisation of tumours in patients with testicular or ovarian cancer using two radioiodinated monoclonal antibodies to placental alkaline phosphatase. *Br. J. Radiol.*, **59**, 117-125.

EVAN, G.I., LEWIS, G.K., RAMSAY, G. & BISHOP, J.M. (1985). Isolation of monoclonal antibodies specific for human c-myc proto-oncogene product. *Mol. Cell. Biol.*, **5**, 3610-3616.

FRAKER, P.J. & SPECK, J.C. (1978). Protein and cell membrane iodinations with sparingly soluble chloramide 1, 3, 4, 6-tetrachloro-3a, 6a-diphenyl-glycoluril. *Biochem. Biophys. Res. Commun.*, **80**, 849-857.

GEORGE, A.J.T., TITUS, J.A., JOST, C.R., KURUCZ, I., TEREZ, T., ANDREWS, S.M., NICHOLLS, T.J., HUSTON, J.S. & SEGAL, D.M. (1993). Single chain Fv directed cellular cytotoxicity (submitted).

GLOCKSHUBER, R., MALIA, M., PFITZINGER, I. & PLUCKTHUN, A. (1990). A comparison of strategies to stabilise immunoglobulin Fv-fragments. *Biochemistry*, **29**, 1362-1367.

HIRD, V., VERHOEYEN, M., BADLEY, R.A., PRICE, D., SNOOK, D., KOSMAS, C., GOODEN, C., BAMIAS, A., MEARES, C., LAVENDER, J.P. & EPENETOS, A.A. (1991). Tumour localisation with a radioactivity labelled reshaped human monoclonal antibody. *Br. J. Cancer*, **64**, 911-914.

HUSTON, J.S., LEVINSON, D., MUDGETT-HUNTER, M., TAI, M.-S., NOVOTNY, J., MARGOLIES, M.N., RIDGE, R.J., BRUCCOLERI, R.E., HABER, E., CREA, R. & OPPERMANN, H. (1988). Protein engineering of antibody binding sites: recovery of specific activity in an anti-digoxin single-chain Fv analogue produced in *Escherichia coli*. *Proc. Natl Acad. Sci. USA*, **85**, 5879-5883.

JAIN, R.K. (1990). Physiological barriers to delivery of monoclonal antibodies and other macromolecules in tumours. *Cancer Res.*, **50**, 814-819.

KENNEL, S.J., FALCIONI, R. & WESLEY, J.W. (1991). Microdistribution of specific rat monoclonal antibodies to mouse tissues and human tumour xenografts. *Cancer Res.*, **51**, 1529-1536.

LAEMMLI, U.K. (1970). Cleavage of structural proteins during the assembly of the head of bacteriophage T4. *Nature*, **227**, 680-685.

MARAVEYAS, A. & EPENETOS, A.A. (1991). An overview of radioimmunotherapy. *Cancer Immunol. Immunother.*, **34**, 71-73.

MARKS, J.D., HOOGENBOOM, H.R., BONNERT, T.P., McCAFFERTY, J., GRIFFITHS, A.D. & WINTER, G. (1991). By-passing immunisation. Human antibodies from V-gene libraries displayed on phage. *Mol. Biol.*, **222**, 581-597.

McCAFFERTY, J., GRIFFITHS, A.D., WINTER, G. & CHISWELL, D. (1990). Phage antibodies: filamentous phage displaying antibody variable domain. *Nature*, **348**, 552-554.

McCARTNEY, J.E., LEDERMAN, L., DRIER, E.A., CABRAI-DENISON, N.A., WU, G.-M., BATORSKY, R.S., HUSTON, J.S. & OPPERMAN, H. (1991). Biosynthetic antibody binding sites: development of a single chain Fv model based on anti-dinitrophenol IgA myeloma MOPC 315. *J. Prot. Chem.*, **10**, 669-683.

MILENIC, D.E., ESTEBAN, J.M. & COLCHER, D. (1989). Comparison of methods for the generation of immunoreactive fragments of a monoclonal antibody (B72.3) reactive with human carcinomas. *J. Immunol. Methods*, **120**, 71-83.

MILENIC, D.E., YOKOTA, T., FILPULA, D.R., FINKELMAN, M.A.J., DODD, S.W., WOOD, J.F., WHITLOW, M., SNOY, P. & SCHLOM, J. (1991). Construction, binding properties, metabolism, and tumor targeting of a single-chain Fv derived from the pancarcinoma monoclonal antibody CC49. *Cancer Res.*, **51**, 6363-6371.

ORLANDI, R., GUSSOW, D.H., JONES, P.T. & WINTER, G. (1989). Cloning immunoglobulin variable domains for expression by the polymerase chain reaction. *Proc. Natl Acad. Sci. USA*, **86**, 3833-3837.

SAVAGE, P., SO, A., SPOONER, R.A. & EPENETOS, A.A. (1993). A recombinant single chain antibody interleukin-2 fusion protein. *Br. J. Cancer*, **67**, 304-310.

SCATCHARD, G. (1949). The attractions of proteins for small molecules and ions. *Ann. N.Y. Acad. Sci.*, **51**, 660-672.

SCHROFF, R.W., FOON, K.A., BEATTY, S.M., OLDHAM, R.K. & MORGAN, A.C. (1985). Human anti-murine immunoglobulin responses in patients receiving monoclonal antibody therapy. *Cancer Res.*, **45**, 879-885.

STAUCH, K.L., JOHNSON, K. & BECKWITH, J. (1989). Characterization of degP; A gene required for proteolysis in the cell envelope and essential for growth of *Escherichia coli* at high temperature. *J. Bacteriol.*, **171**, 2689-2696.

STEWART, J.S.W., HIRD, V., SNOOK, D., SULLIVAN, M., HOOKER, G., COURTENAY-LUCK, N., SIVALOPENKO, G., GRIFFITHS, M., MYERS, M.J., LAMBERT, H.E., MUNRO, A.J. & EPENETOS, A.A. (1989). Intraperitoneal radioimmunotherapy for ovarian cancer. Pharmacokinetics, toxicity and efficacy of I-131 labelled antibodies. *Int. J. Radiat. Oncol. Biol. Phys.*, **16**, 405-413.

TOOLAN, H.W. (1954). Transplantable human neoplasms maintained in cortisone treated laboratory animals: HS1, H.Ep.1, H.Ep.2, H.Ep.3, and H. Emb.Rh.1. *Cancer Res.*, **14**, 660-666.

TRAVERS, P. & BODMER, W.F. (1984). Preparation and characterisation of monoclonal antibodies against placental alkaline phosphatase and other human trophoblastic determinants. *Int. J. Cancer*, **33**, 633-641.

VAUGHAN, A.T.M., ANDERSON, P., DYKES, P.W., CHAPMAN, C.E. & BRADWELL, A.R. (1987). Limitations to the cell killing of tumours using radiolabelled antibodies. *Br. J. Radiolli*, **60**, 567-578.

VERHOEYEN, M., BRODERICK, L., EIDA, S. & BADLEY, A. (1991). Re-shaped human anti-PLAP antibodies. In *Monoclonal Antibodies; Applications in Clinical Oncology*. (ed). Epenetos, A.A., Chapman & Hall, pp. 34-44.

WARD, E.S., GUSSOW, D., GRIFFITHS, A.D., JONES, P.T. & WINTER, G. (1989). Binding activities of a repertoire of single immunoglobulin variable domains secreted from *Escherichia coli*. *Nature*, **341**, 544-546.

YOKOTA, T., MILENIC, D.E., WHITLOW, M. & SCHLOM, J. (1992). Rapid tumour penetration of a single-chain Fv and comparison with other immunoglobulin forms. *Cancer Res.*, **52**, 3402-3408.